# Remarks on the Blending of Periodical and Continued Fevers

**Published:** 1856-06

**Authors:** Austin Flint

**Affiliations:** Buffalo


### BUFFALO MEDICAL JOURNAL

AND

### MONTHLY REVIEW.

VOL. 12. JUNE, 1856. NO. 1.

### ORIGINAL COMMUNICATIONS.

ART. I.—*Remarks on the blending of Periodical and Continued Fevers.* By Austin Flint, M. D.

I shall preface some remarks on an interesting and important subject, viz: the blending of periodical and continued fevers, by the report of a case of disputed type by W. J. Chenoweth, M. D., of Decatur, Illinois. This report was received some months since by my esteemed friend and colleague in the University of Louisville, Professor Rogers, and was accompanied by a request that it be also submitted to my examination. It was designed for publication if deemed advisable. The writer is a young practitioner of much promise. I may add that its publication, with remarks, is at the suggestion of Professor R. The reader will perceive that the case was the occasion of a difference of opinion among a number of physicians who visited the patient during the progress of the disease. The difference was purely one of opinion, and Dr. Chenoweth states in a postscript, that having read the report to Dr. Trowbridge, who saw the patient in consultation oftener than the other medical gentlemen, he expressed satisfaction with it as giving a fair history of the case, although differing from Dr. C. as regards the nature of the febrile affection.

### *A Case of Fever of Disputed Type.*

"The following is one of a class of cases which have occurred in this town and neighborhood, and which have been called remitting fever by some of our physicians, and by others, typhoid. Those of us who believe the disease to be remitting fever treat it with quinine, while those calling it typhoid fever prescribe opiates and stimulants. And inasmuch as the *name* of the disease carries with it different views of its pathology, and will almost necessarily govern the treatment, however guarded we may be, it may be useful to report a single case, so that physicians living in miasmatic districts may be led to give an opinion as to the disease and its treatment. We believe that the importance of being able to diagnose like cases is a sufficient excuse for laying it before the profession.

Sept. 23d, 1855, Rev. Mr. C. was attacked with a chill, followed by a fever. The chill and fever returned on the following day, and for three or four nights following he had "night sweats."

Sept. 30th he again had a chill. The next day he took ten grains of quinine, by the advice of a physician, and in a few days a dose of purgative pills. He had no return of the chill but did not feel well.

Oct. 7th. I was called to see him. I found him (in the forenoon) with a pulse 80 per minute; cool skin; eyes and surface of the body without capillary congestion; mind clear; bowels moved once the day before without medicine.

I ordered 12 grains of quinine to be divided into three doses, one to be given every three hours.

5 o'clock, P. M. Pulse 90; surface of body hot; cheeks flushed; restless and wakeful.

Directed to wash him in cool water.

Oct. 8th, A. M. Learned that he was awake until after twelve o'clock, when he fell asleep and rested until about seven. Pulse 78; much the same appearance as on preceding morning.

Ordered quinine as on yesterday.

P. M. No apparent change since the same hour on the previous day.

Oct. 9th, 9 o'clock, A. M. Has been up for half an hour and has just got to bed. His breathing is a little more labored, but otherwise symptoms as on yesterday. His tongue in the forenoon has been moist, in the afternoons dry. It has been covered with a white fur, and has had a red spot at the tip. He has had liquid stools once or twice daily.

I did not see him in the afternoon of the 9th, but learned that although the paroxysm returned as usual at one o'clock, he was not quite as restless.

Oct. 10th. Very little difference in the symptoms from those present on previous days in the forenoon.

No treatment.

6 o'clock, P. M. Pulse 90; quite restless, (throwing his arms about and changing his position in bed); skin hot; had been dozing a little and imagined he had a body of soldiers at his command, and gave them orders.

Oct. 11th. I called in my partner, Dr. Trowbridge. Pulse 84; breathing regular, but labored; tongue coated and dry; eyes clear; a slight defined redness on both cheeks; skin cool; has had as many as three or four brown colored evacuations during twenty-four hours.

Dr. T. thought the case was probably typhoid fever, but agreed to let him take 15 grains of quinine, divided into three doses. The medicine was left, but as the patient refused to take it, he had no treatment.

P. M. Pulse 96; skin hot and dry; stupid and restless; face sallow; tongue red in the middle.

At Dr. T.'s request we gave him brandy in small quantity, with orders to discontinue it if he was more restless, and substitute Dover's powder, three grains every two hours.

Oct. 12th, A. M. We learned that he had taken the three Dover's powders before he obtained sleep; but as this was at midnight, and he generally fell asleep and rested until morning, we did not know what credit to give to the medicine.

Dr. King was called in consultation, Oct. 13th, and advised to give quinine, but it was not given on account of difference of opinion, until the next day. The symptoms were about the same as on the 12th, except that about daylight he is reported to have sweat.

Oct. 14th. Drs. King and Kellar saw him with Dr. T. and myself. Little, if any, difference in symptoms.

Dr. Kellar proposed to give him valerianate of quinine, which was agreed to by Dr. King and myself, thinking that the difference between that and the sulphate would not warrant us in declining to concur in the proposal. We ordered 5 grains three times a day.

Oct. 15th, 5 o'clock, P. M. Pulse 80; skin hotter than natural; somewhat deaf; drowsy; talking in his sleep; tongue red in spots, where the coating has fallen off; vomited once, water and bilious matter.

Oct. 16th, 9½ o'clock. Pulse 72; has been sweating for half an hour; two rhubarb colored motions since yesterday.

6 o'clock, P. M. Pulse 84; skin dry and hot; paroxysm of fever returned at four instead of one o'clock as previously.

Oct. 17th, 8½ o'clock. Pulse 78; eyes look well, no disposition to stupor; bowels moved once; a slight appearance of sordes on the teeth.

No medicine.

10½ o'clock, A. M. We were sent for under the impression that he had a chill. Mrs. C. had found his hands and feet cold, finger nails blue, and that he was shivering; when we arrived his skin was rather hot, but he drew the bed clothes around him, and was evidently cold. This chill (or as Dr. T. preferred to think, nervous tremor,) lasted three-quarters of an hour. He then threw off part of the bed clothes, and appeared to be quite warm.

6 o'clock, P. M. Pulse 72; mind clear.

In consultation I declined to give brandy, and persisted in giving him 20 grains of the sulphate of quinine (divided into three doses) between twelve o'clock at night and daylight. He had taken on the 14th, 15th and 16th, fifteen grains of the valerianate each day.

Oct. 18th, 9 o'clock, A. M. In a profuse perspiration; pulse 72; mind clear; bowels moved once since yesterday; tongue dry; (he has kept his mouth open, asleep and awake, during his illness.)

3 o'clock, P. M. Not sweating; otherwise as in forenoon.

Oct. 19th, A. M. Pulse 72; mind clear; bowels not moved since yesterday; tongue moist, covered with a light fur; says he is a good deal better.

Oct. 20th, P. M. Had a slight paroxysm of fever at one o'clock.

Nov. 1st. Has been convalescent since I last saw him, and is now able to be up half of the day; wishes food, and complains only of muscular debility.

He has had a small red pimple on the neck, elevated above the surface, and disappearing under pressure, another under the right nipple, a third a little to the right of the median line over the stomach, and between these last, two or three not so well defined. His wife says that mosquitoes have been abundant, and also that there have been fleas about his bed. I could not say that they were not the characteristic rose spots of typhoid fever.

We have been looking at the case from different stand-points, and have called in other counsel, (as the minutes will show). One of them, Dr. King, saw him several times. He has practiced in this town and neighborhood for sixteen years. He agrees with me. Dr. Kellar, who has practiced here for three years, and Dr. McBride, just here from Ohio, (neither of them saw the case but once,) unite with Dr. Trowbridge in calling it a case of typhoid fever. As the case was under my care, and as Dr. King concurred with me in opinion, I persisted, as the notes show, in giving quinine in liberal doses. Dr. Kellar preferred the valerianate, and we therefore gave it. Drs. Trowbridge and McBride declined giving the treatment their approbation.

The grounds for calling the disease typhoid fever were—

1st. Its continuance after the use of quinine, on the 7th and 8th—assuredly not an uncommon thing in remitting or even intermitting fever.

2d. The tongue was coated and dry in the middle. Dr. Flint does not think that the tongue furnishes any positive criterion of the disease.

3d. He had diarrhœa. This is not an uncommon event in ague, and is common in remitting fever, after the use of purgative medicine.

4th. On the 17th there was a slight appearance of sordes, a symptom acknowledged to exist in the typhoid condition occurring in remitting fever.

5th. There was capillary congestion. This was more intense in the afternoon, and presented a dull but defined appearance. His eyes were always clear, until convalescence was established, and then never congested except after sleep.

6th. He was stupid. This was not noticed until on the forenoon of the 12th; he had then been in bed for six or seven days, and the disease had evidently increased, and we may easily account for the stupor by congestion of the brain.

7th. He was deaf. He had taken a good deal of quinine.

8th. He had red spots on his neck and abdomen. My reasons for not believing they were the characteristic spots of typhoid fever are hinted at in the report of the case.

The reasons for believing the case to be remittent fever are the following:

1st. The attack was preceded by intermittent paroxysms, recurring regularly on the seventh day after it was arrested, as is common in ague.

2d. There was a regular exacerbation, commencing at noon and lasting until midnight. Oct. 7th, 8th and 9th, there was almost, if not quite, an intermission in the forenoon.

3d. The fever ended with a chill.

4th. There was no epistaxis.

5th. There was no tympanites."

W. J. Chenoweth.

Decatur, Ill., Nov. 5th, 1855.

The following note, written subsequently to the following report, has an obvious bearing on the question as to the diagnosis:

"Dear Dr.: The person whose case I recently reported to you, moved on Saturday last, Nov. 10th, to a new house, freshly plastered, (the room in which he slept was dry, but the next room was quite wet). On Sunday he ate a small quantity of milk, (his appetite was good,) and in about half an hour he was seized with considerable pain, which he referred to the head of the colon. Injections were given, and two motions produced. He was then given opiates, but the pain continued. On Tuesday evening his bowels were tympanitic, and on Wednesday he died.

I suppose his death was not connected with his first attack as a sequence, but I think it necessary to mention the fact. He was able to walk about, had an excellent appetite, and bid fair to be entirely healthy, but was cut off at a time when hope had well nigh resulted in certainty.

Respectfully, W. J. Chenoweth.

Decatur, Nov. 15, 1856.

Remarks.—Having introduced the foregoing report, an opinion respecting the diagnosis will be expected by the reader as well as the reporter.

I need not say that, in discriminating between diseases which may have many points in common, the data contained in a written description, however complete this may be, are much less satisfactory than the evidence afforded by personal observation. The account of the case, as given by Dr. Chenoweth, was undoubtedly prepared with a disposition to state the facts fairly, and in a truth-seeking spirit.

The circumstances, however, under which the phenomena were observed, and the history related, are such as to render it extremely difficult to divest the mind altogether of bias, and therefore the confidence of the author in the correctness of the position which he was led to take early in the progress of the disease, must not be taken into account in forming an impartial judgment concerning it. There are certain points on which it were to be desired that the report had been more full and explicit, viz.: the condition of the abdomen as respects meteorism, tenderness, and gurgling; the state of the mind; the presence or absence of cough, and the bronchial rales; the period when the spots were observed, and a more minute description of their physical characters.

Judging from the symptoms detailed, and taking into view the occurrence of fatal peritonitis, probably from intestinal perforation, during convalescence,

I must think that the evidence decidedly preponderates in favor of the conclusion that the disease was typhoid fever. It is much to be regretted that the autopsical appearances could not have been ascertained. These, in all probability, would have sufficed, in connection with the *ante-mortem* history, to determine positively the diagnosis.

Occurring, however, in a malarious district, the case presented certain symptomatic events which belong to periodical fever, and which rarely enter into the symptomatology of typhoid fever as observed in parts of the country where intermitting fevers do not prevail. I refer more especially to the recurring febrile paroxysms preceded by chills, which characterised the early part of the history of the case, and the occurrence of a single paroxysm at the termination of the disease. We have then, in this case, apparently an intermingling of the events pertaining to both typhoid and remitting fever; and Dr. Chenoweth states that the case was one of a class of cases observed in that town and neighborhood, which were regarded by some of the physicians as cases of remitting, and by others typhoid fever. Now, is it not probable that there, and in other situations where periodical fevers prevail to a greater or less extent, the two forms of fever, viz., periodical and continued, may be blended, giving rise to a hybrid affection, the phenomena of either species being manifested in different cases in varying proportions? It is with reference mainly to this question that I have introduced this case of disputed type of fever, and to this question the remainder of my remarks will be devoted.

With the present amount of positive knowledge bearing upon it, this is a speculative question. We have not data on which to base a definitive answer. The question can only be answered definitively by historical facts, which it will require not a little time and labor to collect. We are not to prejudge the result of analytical investigation by conclusions which may be hypothetically rational. Nevertheless, here, as in other instances, a discussion professedly on rational or speculative grounds is legitimate, and may be useful by exciting and guiding the researches which will either confirm or disprove the suppositions.

Let us clearly understand what is involved in the hypothesis of the blending of periodical and continued fevers. It is a matter of common observation, that in remitting fevers the occurrence of remissions frequently ceases after a time, and the febrile movement becomes continuous. It is not, however, on this account, regarded as nosologically transformed into continued fever. Practitioners, it is true, often speak of remitting terminating in typhoid fever. But this is a loose mode of expression, which has given rise to not a little confusion. The remitting fever puts on more or less of the external characters which belong to typhoid fever; or, in other words, the patient lapses into a typhoid condition common to a variety of affections. This is all that can with propriety be said, and yet the pertinacity with which many who are not over nice in pathological distinctions, insist on the *conversion* of the one form of fever into the other, is a significant fact to which I shall have occasion presently to refer.* The two forms of fever are, in fact, reckoned distinct genera of the *pyrexiæ.* It may be logically, although not demonstratively proved that each proceeds from the introduction into the system of a special cause; that this morbific agent, or poison, in either instance, is capable of producing a certain definite series of morbid results, which, in the one case, give rise to the characteristic symptomatic phenomena of periodical, and, in the other case, to those of continued fever. The two genera are, therefore, considered as essentially distinct from each other. The poison improperly called *marsh miasmata* is capable of producing the species of fever called intermitting and remitting, and these only; while the special cause of typhoid fever, be it the matter of contagion, or not, will give rise to the species of fever last named, and none other. Now the question is, may these two different morbific agencies act in conjunction within the organism, the processes peculiar to each going on simultaneously, and consequently giving rise to an union of the symptomatic phenomena peculiar to each?

The affirmative answer to this inquiry by no means involves a pathological absurdity. The old doctrine that two diseases cannot co-exist in the same place and time within the body, is now obsolete. Observation has abundantly established that two essentially distinct fevers may run their respective courses simultaneously. Scarlet fever has repeatedly been known

---

* The *conversion,* as distinguished from the *blending* of fevers, is a point which I shall not discuss. The reader is referred to an able article entitled the "blending and conversion of types in fever," from the pen of Prof. Dickson, of Charleston, S. C., contained in the Transactions of the American Medical Association, Vol. V., 1852. Prof. D. shows very clearly the improbability of a conversion of one species of fever into another, in the literal sense of that term. Two diseases, so special in their character, and so distinct as regards their phenomena and their causation as different species of essential fevers, in a certain sense become merged into each other by being blended, but it is not likely that either in reality parts with its individuality, or, in other words, that an actual metamorphosis occurs of the one into the other.

to be thus associated with measles and with small-pox.* The supposition, then, that periodical and continued fevers are capable of being blended, is sustained by analogy.

The special causes of periodical and continued fevers are alike impalpable, inappreciable. In the present state of medical science we can only study their pathological effects. We argue for the distinct specific character of these causes from the uniformity and peculiarity of the effects. This being so, the evidence in behalf of the blending of the two diseases must consist in the union, under certain circumstances, of the symptomatic characters of the two kinds of fever. I have already alluded to the fact so well known to physicians who reside in districts termed malarious, that cases of remitting fever frequently not only fail to preserve during the career of the disease remissions, but present many of the distinctive traits of typhoid fever; and, hence, it appears to the medical observer to be a matter of common sense that the former undergoes a *conversion* into the latter. Is it not reasonable to suppose that in these cases there is actually a combination or blending of the two diseases, albeit the diagnostic criteria by which they are discriminated from each other are sufficiently constant and reliable to enable the practitioner to make the distinction practically in the vast majority of cases?

I am disposed to advance a step beyond an affirmative answer to this question, and to ask whether it be not a rational view of remitting fever to regard it as always involving a blending of the intermittent and the continued. Why do we have in malarious districts, in certain instances, remitting instead of intermitting fever? It is not owing to the greater abundance of the so-called malarious poison, or its greater virulence, as was imagined by Dr. Eberle, for intermitting fever may be as pernicious, in other words, as severe and fatal as remitting fever, and it is fair, other things being equal, to measure the quantity and potency of the special cause by the degree of the specific effects. The specific effects of the malarious poison are intermittent febrile paroxysms, more or less complete and intense recurring at regular intervals. In remitting fever an additional pathological element appears to be involved—two separate affections, viz., a continued and a paroxysmal fever, are united. The disease is a hybrid.

This theory is favored by an inference from a well known fact, which I

---

* For a collection of facts, from different sources, in support of this statement, the reader is referred to a note in Gregory on the Eruptive Fevers, by the American editor, Dr. Buckley, page 344.

do not recollect to have ever seen cited for that purpose. The fact to which I allude is the indigenous development of typhoid fever, more or less abundantly, in different portions of our country, directly intermitting fever disappears, and the infrequency or absence of well marked cases of the former so long as the latter form of fever prevails. This fact has been observed in the locality in which I now write by those who have resided here for the last twenty or more years, and it is a fact which has arrested the attention of observing practitioners in various sections.* Now is it probable that the special cause producing typhoid fever is not developed, or is deficient, in miasmatic districts precisely so long as periodical fever prevails, and that, as a rule, it starts into existence or becomes more abundant just as the latter form of fever disappears? Essentially distinct as are the two poisons, why should the one, in this way, supplant the other? Is it not almost absurd to suppose that the *typhoid* poison, as it were, obsequiously stands aside for *marsh miasmata*, and patiently awaits the departure of the latter before presuming to take its proper place, and assert its rank among the grand morbific agencies of nature! On the other hand, is it not more reasonable to suppose that in districts at one time abounding in periodical fevers, in which subsequently typhoid fever becomes the prevalent form of febrile disease, the special causes of both were at the same time indigenous, and that the association of the two poisons in different relative proportions, gives rise to an union of intermittent and typhoid fever, the phenomena of either prodominating according to the relative quantitative proportion of the efficient causes?

The speculative character of these remarks was conceded at the outset. The blending of periodical and continued fevers, either occasionally in cases of the former, which exhibit in a marked degree more or less of the characters belonging to the latter, or uniformly in cases of remitting as distinguished from intermitting fever, is to be established, or otherwise, by evidence more solid than that pertaining to the considerations just adduced, and others of a kindred character. These considerations claim attention only as suggestive of an interesting and important subject for scientific investigation. What are the requisites for an investigation directed to the points which have been raised? A few words with reference to this inquiry.

Bearing in mind our ignorance of the special causes involved in the production of both forms of fever—of their nature and origin as well as

---

* For a collection of testimony relative to this point, the reader is referred to Bartlett's Treatise on Fever. Edition of 1847, page 108 *et seq.*

the primary and essential morbid processes to which they give rise when they are introduced within the organism, it is plain that the supposition of their conjunctive operation is to be proved or disproved by the analytical study and comparison of their appreciable phenomena, that is, the symptomatic characters of the two kinds of fever. The data for this method of investigation are yet to be acquired. The natural history of continued fever, so far as concerns the species now known as typhus and typhoid, is pretty well ascertained. These fevers have been carefully studied by means of the analysis of recorded cases in different countries. As much can not be said of periodical fever, especially the species or variety called remitting. The natural history of this fever can not be considered as satisfactorily settled on the basis of that of the two species of continued fever just mentioned. Great as are the opportunities in various parts of this country, and in other countries, to collect recorded cases of remitting fever for analysis, it remains to accomplish for this disease what the labors of Louis, Gerhard and others, have done for typhus and typhoid fever. Something has been contributed toward an end so desirable by Stewardson * and Alfred Stillé,† but the materials gathered by these distinguished physicians, although valuable, are not sufficient. What a tempting field is here open to the clinical observer and historian! But of how many diseases may this be said! "Truly the harvest is great, but * * * *"

The results of the analyses of different collections of recorded cases of remitting fever in different situations, including districts in which typhoid fever is seldom or never seen, and those in which it has become more or less prevalent, will establish the points pertaining to its natural history which distinguish the former from the latter; and by bringing these results together and comparing them with each other, it will be seen whether remitting fever maintains uniformly certain characters essentially distinct, or whether in proportion as cases of periodical fever disappear, and cases of continued fever succeed, the symptomatic phenomena of remitting fever manifest a transition to typhoid, showing a gradual predominance of the characters of the latter until those of the former are finally lost.

According to the general impressions of observing practitioners who have seen more or less of the disease, the descriptions by various authors,

---

*Analysis of twenty cases of remitting fever in the Am. Jour. of Med. Sciences, April, 1841, and April, 1842.

† Analysis of ten cases of remitting fever recorded by Dr. ———, of Baltimore, by Alfred Stillé, M. D., of Philadelphia, April, 1846.

based on unrecorded experience, together with the results of analytical investigation (as yet far too limited) of recorded cases, certain differential characters of remitting and typhoid fever are generally sufficiently marked to serve as diagnostic criteria. It is, however, admitted that in a certain proportion of instances a positive discrimination is not easily made, especially in cases not observed from the commencement of the disease; and it is perhaps not incorrect to say that, in certain situations it is oftener difficult to determine whether the disease be typhoid or remitting fever than to distinguish typhoid from typhus fever, in places where the two latter diseases prevail. As already stated, it is a matter of common observation that the symptoms generally characterised as *typhoid*, such as low, muttering delirium, a dry, furred tongue, sordes, etc., are not infrequently developed in the course of remitting fever. The points to be settled are, to what extent are the characters considered as distinctive of each form really entitled to be regarded in this light, and of the characters which are truly distinctive, what significance or importance do they possess as representatives of the essential nature of the disease. Take for example the abdominal symptoms which belong to the natural history of typhoid fever, viz., diarrhœa, meteorism, tenderness, gurgling. These are undoubtedly wanting, as a rule, in cases of remitting fever; but are they not present in a certain proportion of cases, and if so, in what proportion? Again, complementarily to the inquiry just made, are the intestinal lesions supposed to be characteristic of typhoid fever, ever found in cases which, irrespective of their existence and of the presence of the symptomatic phenomena associated with them, would be entitled to be called cases of remitting fever? A statement to that effect is positively made by a distinguished teacher and writer;* can it be substantiated by evidence adduced by the analyses of recorded cases? The same interrogatories may be applied to the eruption, and to other diagnostic events which are of more or less significance and importance in their relations to the essential or special nature of the disease. When these points are settled, as they may be, and as they alone can be by the accumulated results of numerous analyses of recorded cases, then we shall be prepared to bring logical proof for or against the doctrine that remitting fever, either occasionally or habitually, involves a blending of the special causes and essential pathological processes pertaining to the periodical and continued fevers.

---

* Prof. Dickson, in his paper on the "Blending and Conversion of Types in Fever," already referred to.

In the mean time, indulging, as we may do, theoretical views, (provided they are not permitted to engender pre-conceptions which will blind the perception of truth,) this doctrine is sustained by cogent considerations, and explains satisfactorily well known facts. Variation in the relative proportion of the *periodical* and the *continued* causative and pathological elements, serves to account for certain palpable differences in cases of remitting fever. We can understand that in proportion as the former elements predominate, the character of the disease will approximate to that of intermittents; the febrile exacerbations will approach to paroxysms, and the career of the disease will be arrested by anti-periodic remedies. On the other hand, the predominance of the latter elements will proportionately give rise to the phenomena which are characteristic of typhoid fever, in addition to continuity in the febrile movement. These are the cases which resist the sulphate of quinia in large doses, and which sometimes perplex even the accomplished diagnostician.

In the same way we may account for a fact which might be cited as tending strongly to sustain the doctrine. I refer to the prevalence of remitting fever in malarous districts during seasons when cases of intermitting fever are comparatively unfrequent, and *vice versa*. It is well known that the two forms of periodical fever (intermitting and remitting) do not observe any law of relative proportion in their concurrence. In one year intermitting fever may be rife, and remitting fever be rarely observed, while in another year remitting fever is abundant and intermittents not unusually prevalent. Now, is this fact consistent with the notion that remitting fever involves the same special cause, and that only, which produces intermitting fever? Is it not, on the other hand, more consistent with the supposition that two special causes are conjoined, viz., that which is called marsh miasma and the typhoid poison? Adopting this supposition, when remitting fever preponderates, the typhoid poison has the ascendency. Under these circumstances, were the district not malarious, there would be an endemic of pure typhoid fever. But when intermitting fever is the prevailing form, the special cause of a purely periodical fever abounds, unattended by the typhoid poison.

In conclusion, the doctrine in behalf of which these few discursive remarks are offered, simply assumes that the special cause of typhoid fever* is not restricted, geographically, to territory free from periodical fevers, but,

* I limit this statement to typhoid fever, inasmuch as this is the species of continued fever chiefly indigenous in this country.

existing in miasmatic districts, is associated in its morbid manifestations to a greater or less extent, with the phenomena due to the special cause of periodical fever, and, thus associated, gives rise to remitting fever, which is therefore a hybrid affection. This, in reality, is only an extension to continued fever of a fact to which the experience of all practitioners residing in malarious districts will testify, viz.: that, in general, the various affections originating in these districts are liable to receive important modifications from the conjunction of the malarious poison.

Buffalo, May 1, 1856.

